# Error Compensation without a Time Penalty: Robust Spin-Lock-Induced Crossing in Solution NMR

**DOI:** 10.1021/acs.jpclett.6c00524

**Published:** 2026-03-23

**Authors:** Mohamed Sabba, Christian Bengs, Urvashi D. Heramun, Malcolm H. Levitt

**Affiliations:** School of Chemistry and Chemical Engineering, 7423 University of Southampton, Southampton SO17 1BJ, United Kingdom

## Abstract

A modification of the widely used spin-lock-induced crossing (SLIC) procedure is proposed for the solution nuclear magnetic resonance (NMR) of strongly coupled nuclear spin systems, including singlet NMR and parahydrogen-enhanced hyperpolarized NMR experiments. The compensated-SLIC (cSLIC) scheme uses a repetitive sequence where the repeated element employs two different radio-frequency field amplitudes. Effective compensation for deviations in the radio-frequency field amplitude is achieved without increasing the overall duration of the SLIC sequence. The advantageous properties of cSLIC are demonstrated by numerical simulations and representative experiments.

## Graphical abstract

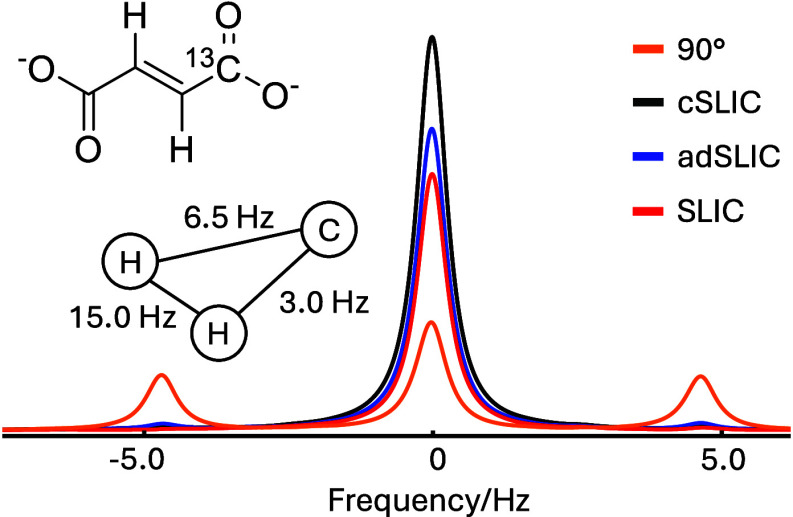

The spin-lock-induced crossing (SLIC) method was introduced by DeVience et al. in 2013 as a simple method for generating a long-lived singlet order for near-equivalent spin pairs in nuclear magnetic resonance.^1^ SLIC involves the application of a resonant radio-frequency (rf) field with the same phase as transverse nuclear spin magnetization. The amplitude of the resonant field is chosen so that the nutation frequency under the rf field matches the *J* coupling between the members of the spin pair

1 $$\omega_{\mathrm{nut}}=\omega_{J}$$

where ω_*J*_ = 2π*J*. When applied to strongly coupled spin-1/2 pairs |ω_Δ_| ≪ |ω_*J*_|, where ω_Δ_ = 2πΔ (and Δ is the chemical shift difference in units of Hz), this matching condition induces a level crossing in a suitable reference frame,^2^ which leads to the coherent evolution of transverse magnetization into nuclear singlet order (SO). Singlet order represents a net difference in populations of the singlet and triplet states of the spin-1/2 pair

2 $$Q_{\mathrm{SO}}=\left|S_{0}\right\rangle \left\langle S_{0}\right|-\frac{1}{3}\underset{m}{\sum }\left|T_{m}\right\rangle \left\langle T_{m}\right|=-\frac{4}{3}I_{j}\cdot I_{k}$$

where |*S*_0_⟩ and |*T*_*m*_⟩ are the singlet and triplet states formed by the two participating spins *I*_*j*_ and *I*_*k*_.^3,4^ In suitable circumstances, singlet order is a “long-lived state”, protected against some common relaxation mechanisms, which may exhibit a decay time constant exceeding 1 h in favorable circumstances.^5^

SLIC has also been used for the selection of desirable NMR^6^ and MRI^7,8^ signals, the estimation of *J*-coupling differences on the order of a few mHz,^9^ chemically informative *J* spectroscopy in low magnetic fields,^10–12^ and parahydrogen-enhanced NMR.^7,13–18^ SLIC may also be used to manipulate multiple-quantum transitions in strongly coupled spin systems.^19–23^

Many applications of SLIC are hampered by its high sensitivity to deviations in the rf field amplitude. This behavior is illustrated by the contour plot in Figure 1a, which shows the amplitude for the transformation of transverse nuclear magnetization (described by the operator *I*_*x*_) into nuclear singlet order (described by the operator *Q*_SO_). The transformation amplitude is plotted as a function of two parameters. The horizontal axis shows the *J*-coupling-normalized resonance offset

3 $$\Omega =\Omega_{\mathrm{rf}}-\frac{1}{2}\left(\Omega_{j}^{0}+\Omega_{k}^{0}\right)$$

where Ω_rf_ is the radio-frequency field frequency and $\Omega_{j}^{0}\mathrm{and}\Omega_{k}^{0}$ are the chemically shifted Larmor frequencies of the two spins. The vertical axis shows the fractional deviation of the rf field amplitude, expressed as a nutation frequency, ω_nut_, from the nominal value $\omega_{\mathrm{nut}}^{0}$ .

4 $$\varepsilon_{\mathrm{rf}}=\frac{\omega_{\mathrm{nut}}-\omega_{\mathrm{nut}}^{0}}{\omega_{\mathrm{nut}}^{0}}$$

The narrow crescent shape traced out by the contours in Figure 1a indicates that the performance of SLIC is very sensitive to the rf amplitude when applied at an exact resonance and also that the resonance offset and rf amplitude errors interact strongly. In general, these are undesirable characteristics. In particular, the high rf field sensitivity implies a degradation in performance whenever the rf field is inhomogeneous over the sample volume, as is often the case. The strong interaction of rf field amplitude and resonance offset impedes the manipulation of spin systems with different chemical shift values and makes it difficult to achieve clean frequency selectivity, as in experiments on multiple-spin systems.^20–24^

**Figure 1:**
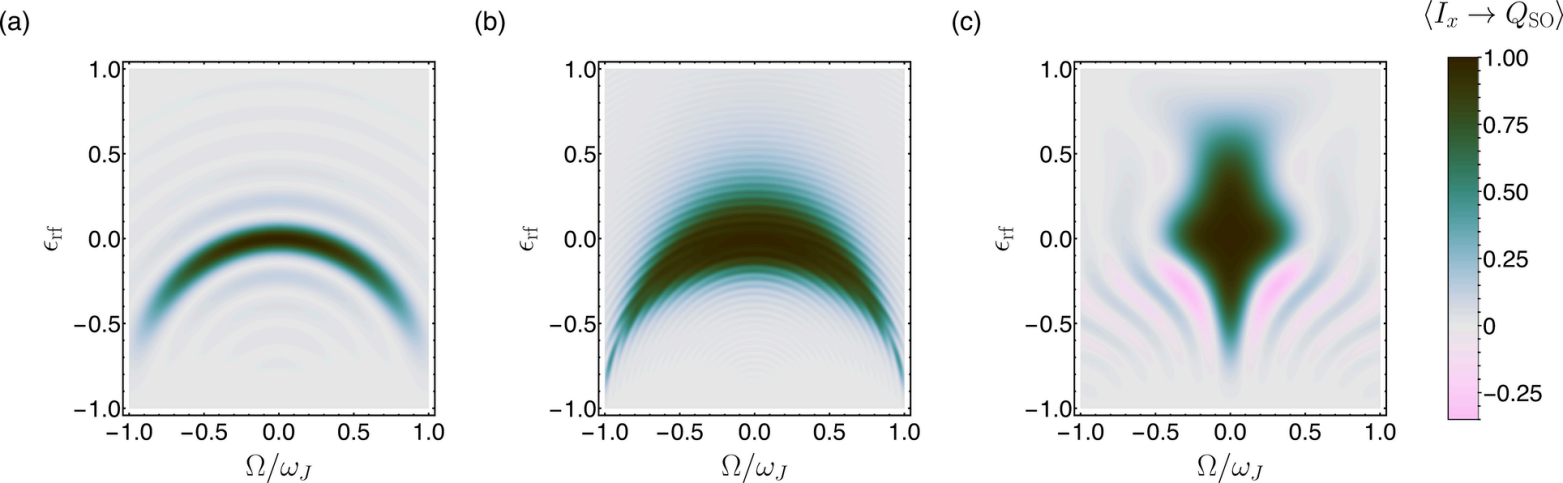
Contour plots for the transformation amplitude of transverse magnetization into singlet order, defined by ⟨*I*_*x*_ → *Q*_SO_⟩ = Tr­{*Q*_SO_ *UI*_*x*_ *U* ^†^}/Tr­{*Q*_SO_ *Q*_SO_}, where *U* represents the propagator for a specific SLIC element. Results are shown for (a) SLIC, (b) adSLIC, and (c) cSLIC against resonance offset (horizontal axis) and deviations in the rf amplitude (vertical axis). All simulations are performed for a two-spin system with *J* = 15 Hz and Δ = 1.9 Hz. The total durations of the elements *T* are 375 ms (SLIC and cSLIC) and 1560 ms (adSLIC), respectively. For cSLIC, the repetition number is *n* = 6 and α = 0.99. For adSLIC, the rf amplitude modulation is given by eq 5 with Δ_max_ = 0.5 and ξ = 0.9.

Several methods have been proposed for rendering SLIC less sensitive to amplitude errors. One popular option is to extend the pulse sequence duration while varying the rf amplitude continuously so as to sweep through a range of matching conditions.^24,25^ This solution is often known as adiabatic SLIC (adSLIC) even though the strict adiabaticity conditions on the spin dynamics are not always met.^25,26^ Simulations for one possible amplitude shape given by Theis and co-workers^24,27^ are shown in Figure 1b, where the specific rf amplitude modulation is given by

5 $$\begin{array}{l} \omega_{\mathrm{nut}}\left(t\right)=\omega_{J}\left[1-\Delta_{\max}\tan\left(x\xi \pi /2\right)/\tan\left(\xi \pi /2\right)\right]\\ x=2t/T-1 \end{array}$$

where the time variable *t* runs from 0 to the total duration *T*. The parameter −1 < Δ_max_ < 1 defines the size and direction of the sweep, whereas 0 < ξ < 1 is a shape parameter, with small values of ξ converging to a linear sweep and large values providing a more inflected shape, with slower amplitude variation in the center of the shape and more rapid amplitude changes toward the ends.

As expected, sensitivity to the rf amplitude is reduced relative to unmodulated SLIC, albeit at the cost of a significantly increased duration, which can lead to increased losses through dissipation, although this is not taken into account in the calculations in Figure 1. In practice, this dissipation sets the ultimate limit on performance enhancement, while, in principle, the robustness of adSLIC can be increased indefinitely by increasing the pulse duration, which is not possible in spin systems that relax rapidly. In the simulation of Figure 1b, a total adSLIC duration of *T* = 500 ms was chosen, approximately 7 times longer than the SLIC sequence, to ensure a fair comparison.

We recently showed that SLIC may also be used for double-quantum excitation in systems of spin-1/2 pairs and proposed a modified SLIC sequence, called compensated SLIC or cSLIC, in order to improve its tolerance to errors.^28^ The cSLIC sequence was shown to provide effective compensation for rf amplitude deviations without an increase in pulse sequence duration relative to the uncompensated SLIC method. Here, we show that the cSLIC sequence is also significantly more robust than uncompensated SLIC in the context of singlet NMR and in contexts such as parahydrogen-enhanced NMR.

The cSLIC sequence involves *n* repetitions of the cyclic element C sketched in Figure 2a, which has the form

6 $$C={\left(\alpha \pi \right)}_{x}^{\mathrm{weak}}-{\left(\alpha 2\pi \right)}_{-x}^{\mathrm{strong}}-{\left(\alpha \pi \right)}_{x}^{\mathrm{weak}}$$

Compared to conventional SLIC, the cSLIC sequence employs two different amplitude levels: a weak rf field and a stronger rf field, which provides a compensatory counter rotation in the center of each repeating element. The nutation frequency of the two weak outer pulses matches the SLIC condition.

7 $$\omega_{\mathrm{nut}}^{\mathrm{weak}}=\omega_{J}$$

By contrast, the strong central pulse is assumed to have a nutation frequency that is much larger than those of the outer pulses, $\omega_{\mathrm{nut}}^{\mathrm{strong}}>\omega_{\mathrm{nut}}^{\mathrm{weak}}$ . The parameter $\frac{1}{2}<\alpha ≲1$ is given by

8 $$\alpha =\frac{\omega_{\mathrm{nut}}^{\mathrm{strong}}}{\omega_{\mathrm{nut}}^{\mathrm{strong}}+\omega_{\mathrm{nut}}^{\mathrm{weak}}}$$

In the limit of an infinitesimally short central pulse $\left(\omega_{\mathrm{nut}}^{\mathrm{strong}}\gg \omega_{\mathrm{nut}}^{\mathrm{weak}}\right)$ , the parameter α tends to 1 and the cSLIC sequence becomes

9 $$\mathrm{cSLIC}\left(\alpha \to 1\right)={\left(\pi \right)}_{x}^{\mathrm{weak}}-{\left(2\pi \right)}_{-x}^{\mathrm{strong}}-{\left(\pi \right)}_{x}^{\mathrm{weak}}$$

where the central 2π pulse has negligible duration. In practice, this limit cannot be reached due to hardware limitations, but it can be approached sufficiently closely.

**Figure 2:**
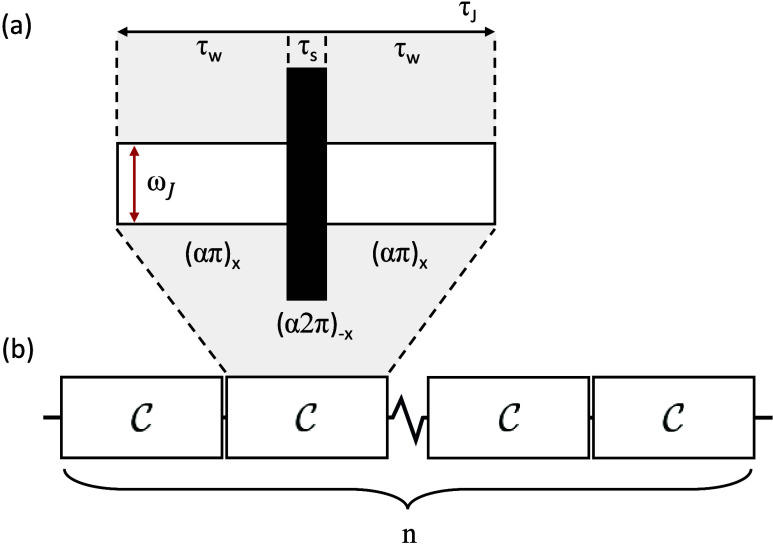
Pulse sequence schematic for cSLIC. (a) Basic element consists of a concatenation of two weak pulses separated by a strong pulse. The weak pulses are of amplitude $\omega_{\mathrm{nut}}^{\mathrm{weak}}=\omega_{J}$ and duration τ_w_ and produce a net rotation of (απ) along the *x* axis. The strong central pulse is of amplitude $\omega_{\mathrm{nut}}^{\mathrm{strong}}>\omega_{J}$ and duration τ_s_ and produces a net rotation of (α2π) along the −*x* axis. The parameter $\frac{1}{2}\leq \alpha ≲1$ is defined by eq 8. The pulse durations are constrained by 2τ_w_ + τ_s_ = τ_*J*_. (b) cSLIC-based singlet excitation consists of $n=\left\lfloor J/\left(\sqrt{2}\Delta \right)\right\rceil$ repetitions of the basic element shown in panel a.

The duration of each cSLIC element is given by τ_*J*_ = |*J*|^–1^ for all values of α. In the absence of relaxation, the singlet-order generation for an ensemble of 2-spin-1/2 systems is maximized when *n* is chosen as follows:^28^

10 $$n=\left\lfloor J/\left(\sqrt{2}\Delta \right)\right\rceil$$

where ⌊*x*⌉ rounds *x* to its nearest integer.

The compensation principle can be illustrated in the limit α = 1, where the duration of the central compensating pulse is negligible compared to the element duration τ_*J*_. The assumption is that the sequence is applied on resonance (Ω = 0). Under these ideal conditions, the two weak SLIC pulses combine to generate a 2π rotation, and the central pulse generates an equal and opposite 2π rotation. When the rf field amplitude of the weak pulses is larger than expected, $\omega_{\mathrm{nut}}^{\mathrm{weak}}=\left(1+\varepsilon_{\mathrm{rf}}\right)\omega_{J}$ , the excess rotation induced by the misset SLIC field is compensated by the equal and opposite excess rotation caused by the misset strong pulse and similarly when the rf field is weaker than nominal. This compensation mechanism assumes that the weak and strong rf fields experience rf amplitude errors in the same proportion. This is the case when both fields are generated by the same radio-frequency coil and when the deviations are caused by spatial variations in the rf field strength. This is the usual experimental situation.

If the central pulse strength does not dominate the outer pulses (α < 1), the net rotations of the inner and outer pulses still compensate each other for any value of the overall rf amplitude, and the duration of each repeating element still matches one period τ_*J*_ of the *J*-coupling frequency. Hence, the basic physics of the level anticrossing^2,14,29,30^ still applies, albeit with slightly reduced state-mixing coefficients. The simulation shown in Figure 1c confirms this analysis for the realistic case α = 0.99. The robustness of the singlet excitation amplitude with respect to rf amplitude deviations is greatly improved at the expense of a somewhat reduced frequency bandwidth. The undesirable interaction between the rf amplitude and resonance offset effects is strongly reduced. The improved error compensation of cSLIC is achieved with the same sequence duration as that of uncompensated SLIC.

## Experimental Details

The experimental performance of the SLIC, adSLIC, and cSLIC sequences was evaluated by performing singlet-mediated heteronuclear polarization transfer experiments on a solution of [1-^13^C]-fumarate in D_2_O. The heteronuclear pulse sequence scheme shown in Figure 3a was used. This has previously been employed to evaluate SLIC and related methods in the context of parahydrogen-enhanced ^13^C NMR.^15^ Although the two ^1^H nuclei in [1-^13^C]-fumarate are chemically equivalent, they have different *J* couplings with the ^13^C nucleus. This *J*-coupling difference effectively takes the role of the chemical shift difference in spin-1/2 pairs,^15,31^ allowing the generation of ^1^H singlet order from ^1^H transverse magnetization when a ^1^H SLIC sequence is applied for a duration *T*_H_. The *J*-coupling difference also allows the conversion of ^1^H singlet order into ^13^C transverse magnetization when a subsequent SLIC sequence is applied on the ^13^C channel for a duration *T*_C_. In the absence of dissipation, the optimal duration of the second SLIC pulse is shorter than that of the first SLIC pulse, by a factor of $\sqrt{2}$ (see ref 15).

**Figure 3:**
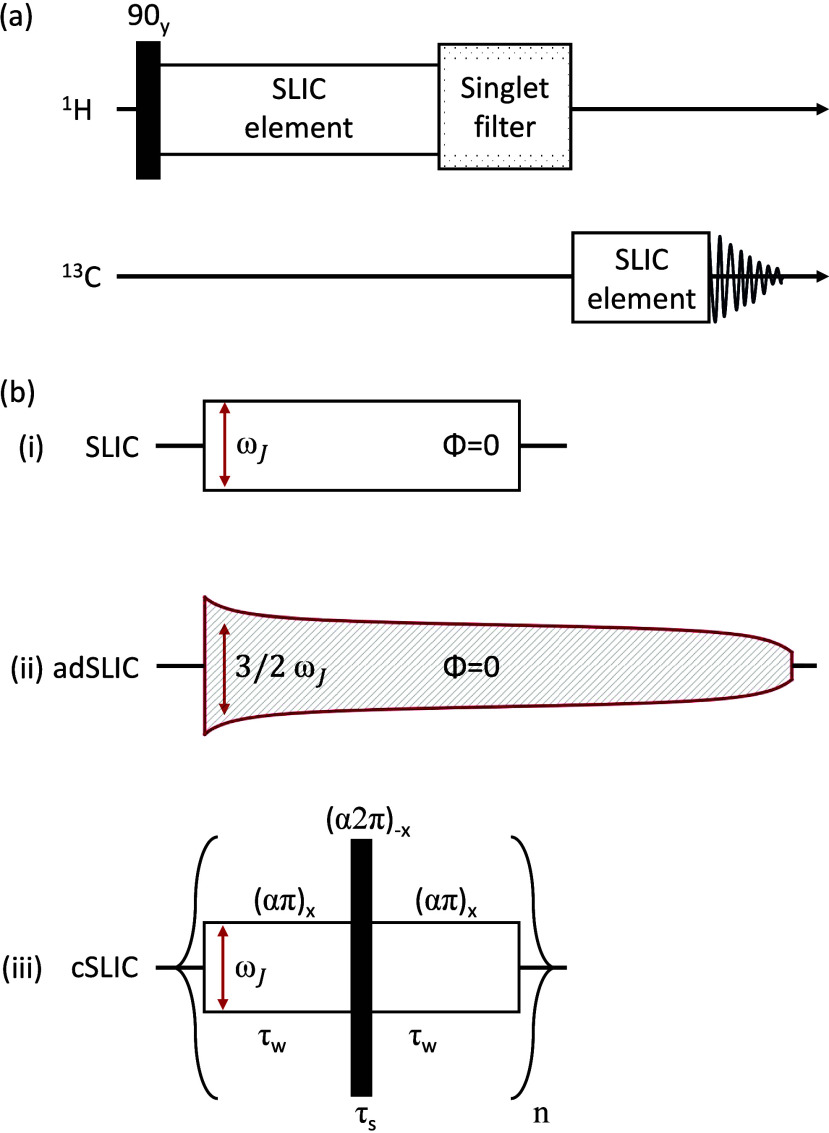
(a) Pulse sequence for heteronuclear polarization transfer from ^1^H to ^13^C, through an intermediate ^1^H singlet state, in systems of two ^1^H nuclei and one ^13^C nucleus, as in Figure 4. An initial 90_*y*_^°^ pulse generates transverse proton magnetization, followed by a singlet preparation element using one of the SLIC variants shown in panel b. After singlet-order preparation, a filter element removes any spurious density-operator terms. A second SLIC variant transforms singlet order into heteronuclear magnetization. (b) SLIC elements used in this work. (i) Basic SLIC element consists of a single *x* pulse with amplitude ω_*J*_. (ii) Adiabatic SLIC consists of an amplitude-modulated *x* pulse following the functional form given in eq 5. (iii) cSLIC element follows the procedure outlined in Figure 2.

Experiments were performed on a 200 mM solution of [1-^13^C]-fumarate in D_2_O at a magnetic field of 9.4 T. The pulse powers were adjusted to provide a nutation frequency of 25.0 kHz on the ^13^C channel and 10.0 kHz on the ^1^H channel, corresponding to 90° pulse durations of 10 and 25 μs, respectively. The pulse sequence in Figure 3a deploys a standard singlet filtration sequence^32,33^ in order to suppress any ^13^C NMR signals that do not pass through ^1^H singlet order between the two SLIC sequences. The experimentally optimized parameters for the sequences were {*T*_H_, *T*_C_} = {524 ms, 370 ms} (SLIC), {*T*_H_, *T*_C_, *n*_H_, *n*_C_, α} = {500 ms, 381 ms, 8, 6, 0.988} (cSLIC), and {*T*_H_, *T*_C_, Δ_max_, ξ} = {1780 ms, 1560 ms, 0.5, 0.9} (adSLIC), and ω_*J*_/(2π) = 15 Hz.

Figure 4 (orange) shows a ^13^C spectrum of [1-^13^C]-fumarate solution generated by a 90° ^13^C pulse applied to the sample in thermal equilibrium. ^1^H decoupling was not applied. The ^13^C spectrum shows a triplet multiplet structure due to the *J* couplings of the ^13^C nucleus to the two fumarate ^1^H nuclei, which are nearly magnetically equivalent due to the large *J*_HH_ coupling, the relatively small difference between the two *J*_CH_ couplings, and the very small difference in chemical shifts between the two ^1^H sites.^15^

**Figure 4:**
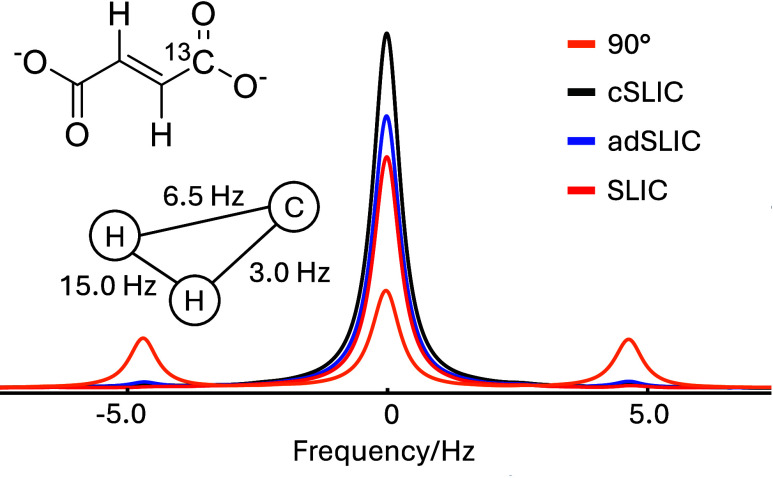
^13^C spectra of [1-^13^C]-fumarate dissolved in D_2_O. The inset shows the chemical structure and *J*-coupling parameters. Fourier transform (orange) of the free-induction decay generated by a single 90° ^13^C pulse applied to a sample in thermal equilibrium. ^13^C spectra for SLIC (red), adSLIC (blue), and cSLIC (black) were obtained using the pulse sequence strategy shown in Figure 3. 0.5 Hz of line broadening was applied to all spectra. The pulse sequence parameters are summarized in the Experimental Details.

Figure 4 (red) shows a ^13^C spectrum obtained by generating ^1^H singlet order using a conventional ^1^H SLIC sequence of duration *T*_H_ = 524 ms, suppressing any other density operator components by a singlet filter sequence and applying a conventional ^13^C SLIC sequence of duration *T*_C_ = 370 ms to generate transverse ^13^C magnetization. The signal intensity is enhanced relative to that in Figure 4 (orange), since the ^1^H singlet order is generated from thermal-equilibrium ^1^H magnetization, which is approximately 4 times larger than that of ^13^C. The outer multiplet components of the ^13^C signal are suppressed, as expected by theory.^15^

Figure 4 (blue) shows a ^13^C spectrum of [1-^13^C]-fumarate obtained under identical conditions but using adSLIC sequences for both of the SLIC elements in Figure 3. The adSLIC elements followed the functional form given in eq 5 and had overall durations of *T*_H_ = 1780 ms and *T*_C_ = 1560 ms, with Δ_max_ = 0.5 and ξ = 0.9 in both cases. The spectrum shown in Figure 4 (blue) was obtained after a sincere attempt to optimize the adSLIC shape parameters on both channels; longer durations led to a deterioration of the performance. In this case, only a moderate improvement is evident over the fixed-amplitude SLIC result in Figure 4 (red).

Figure 4 (black) shows the ^13^C spectrum obtained when using cSLIC for both SLIC elements of the pulse sequence. The parameters used were *T*_H_ = 500 ms, *T*_C_ = 381 ms, *n*_H_ = 8, *n*_C_ = 6, and α = 0.988. The spectrum obtained with cSLIC shows a significantly stronger signal than that achieved with both SLIC and adSLIC.

Figure 5 demonstrates the experimental response of the three methods to rf amplitude variations. In all cases, the amplitudes of the strong and weak pulses were kept in the same fixed ratio. SLIC displays poor compensation against field errors. Deviations as small as ±10% in the rf amplitude reduce the polarization transfer amplitude by ∼50%. adSLIC displays considerably more robustness with respect to rf amplitude deviations, especially on the high-amplitude side. In contrast, cSLIC remains largely unaffected by modest rf field variations, retaining half of its peak efficiency even for fractional errors as large as ±50%. The enhanced amplitude of cSLIC over SLIC and adSLIC, as shown in Figure 4, may thus be attributed to the substantially reduced sensitivity of cSLIC to rf field amplitude variations across the sample volume.

**Figure 5:**
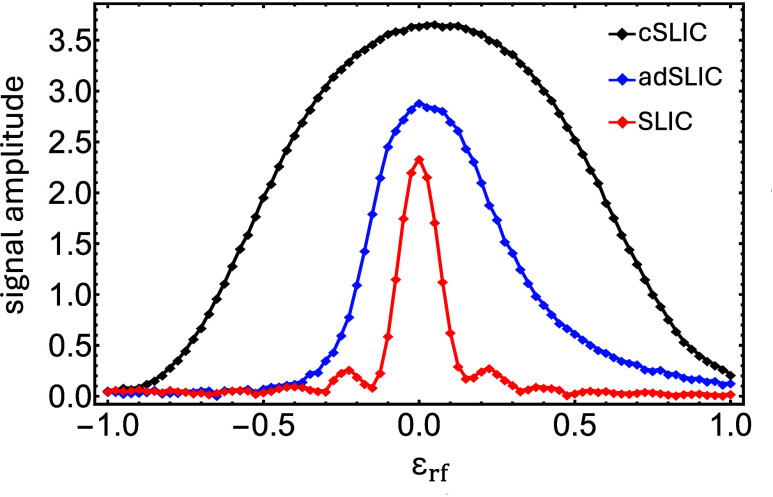
Experimental ^13^C signal amplitude for singlet-mediated heteronuclear polarization transfer in [1-^13^C]-fumarate, as a function of the fractional ^13^C nutation amplitude mismatch, defined in eq 4. Signal amplitudes have been normalized against the amplitude of the central peak in the 90° spectrum.

This work demonstrates that cSLIC provides markedly improved compensation against rf field deviations compared to SLIC and adSLIC, retaining high singlet-order transfer efficiencies even under substantial nutation amplitude mismatches. Although not demonstrated explicitly, this enhanced robustness makes cSLIC particularly attractive for parahydrogen-induced polarization (PHIP) applications, where maximizing heteronuclear polarization transfer is critical for achieving practical levels of hyperpolarization. Importantly, cSLIC achieves these advantages as a relatively low-power method: although the central pulse is referred to here as “strong”, it needs to be only modestly more intense than the conventional SLIC element; typically, around 5 times the SLIC field is sufficient. Counterintuitively, the error compensation is achieved without increasing the overall sequence duration, in contrast to the composite pulse^24^ or optimal control variants of SLIC, which are power-efficient but have a lengthened duration.^34^ The method is also fully compatible with magic-angle (MA) compensation schemes for dipolar field effects, which are important for attaining high molar polarization in PHIP applications.^18,35^ Additionally, as shown in section II of the Supporting Information, the performance of the cSLIC sequence can be further enhanced through supercycling, an option that is not readily available for the other methods and may even be used for DNP. Collectively, these features should make cSLIC a practical tool for enhancing experimental transfer efficiencies in a wide range of NMR experiments exploiting nuclear singlet states and similar phenomena.

## Supplementary Material



## Data Availability

The data that support the findings of this study are available from the corresponding author, Mohamed Sabba, upon reasonable request.

## Notes

Brief details of the theory for multiple use cases of the (c)­SLIC sequence and simulations of supercycled variants of cSLIC (PDF)
