# Supplementary material for: Error Compensation without a Time Penalty: Robust Spin-Lock-Induced Crossing in Solution NMR
Source: J Phys Chem Lett. 2026 Mar 23;17(14):4161–6. doi: 10.1021/acs.jpclett.6c00524 (PMC13071919; doi:10.1021/acs.jpclett.6c00524)
Supplement: Supplementary file 1 [file jz6c00524_si_001.pdf]

Error compensation without a time penalty: robust spin-lock-induced crossing in solution NMR

Mohamed Sabba,[1, a)] Christian Bengs,[1] Urvashi D. Heramun,[1] and Malcolm H. Levitt[1]
Department of Chemistry, University of Southampton, SO17 1BJ, UK

(Dated: March 13, 2026)

[a)]Electronic mail: m.sabba@soton.ac.uk

ORGANISATION OF THE SUPPORTING INFORMATION

The Supplementary Information (SI) is organised as follows. Section I presents the theory of SLIC within the framework of effective Hamiltonian theory, highlighting its connections to a broader class of PHIP and DNP problems, in the spirit of previous references[1,2]. Section II briefly describes supercycling methods used to further improve the uniformity of the cSLIC frequency response.

I. BASIC THEORY OF SLIC

A. Single transition operators

In order to rationalize SLIC and cSLIC by effective Hamiltonian theory, it is appropriate to define single-transition operators[3,4]. A set of single-transition operators for the subspace of the two arbitrary states $|\alpha\rangle$ and $|\beta\rangle$ could be written as:

$$\begin{aligned}
I_x^{\alpha,\beta} &= \frac{1}{2}\left(|\alpha\rangle\langle\beta| + |\beta\rangle\langle\alpha|\right) \\
I_y^{\alpha,\beta} &= \frac{1}{2i}\left(|\alpha\rangle\langle\beta| - |\beta\rangle\langle\alpha|\right) \\
I_\phi^{\alpha,\beta} &= \cos(\phi)\, I_x^{\alpha,\beta} + \sin(\phi)\, I_y^{\alpha,\beta} \\
I_z^{\alpha,\beta} &= \frac{1}{2}\left(|\alpha\rangle\langle\alpha| - |\beta\rangle\langle\beta|\right) \\
\mathbb{1}^{\alpha,\beta} &= |\alpha\rangle\langle\alpha| + |\beta\rangle\langle\beta|
\end{aligned} \tag{1}$$

B. The effective Hamiltonian of SLIC in an AB spin system

The singlet and triplet states of an isolated spin-1/2 pair are defined as follows:

$$\begin{aligned}
|S_0\rangle &= (|\alpha\beta\rangle - |\beta\alpha\rangle)/\sqrt{2}, \\
|T_+\rangle &= |\alpha\alpha\rangle, \\
|T_0\rangle &= (|\alpha\beta\rangle + |\beta\alpha\rangle)/\sqrt{2}, \\
|T_-\rangle &= |\beta\beta\rangle
\end{aligned} \tag{2}$$

And the Hamiltonian of this spin system, H_{AB}, can be expressed like so:

$$H_{\mathrm{AB}} = H_{\Delta} + H_J \tag{3}$$

$$H_{\Delta} = \omega_{\Delta}(I_{1z} - I_{2z})/2 = \omega_{\Delta} I_x^{T_0,S_0} \tag{4}$$

$$H_J = \omega_J I_1 I_2 = \omega_J \left(I_z^{T_0,S_0} + I_{1z} I_{2z} \right) \tag{5}$$

Where $\omega_J = 2\pi J_{12}$ represents the J-coupling and $\omega_{\Delta} = 2\pi\Delta$ represents the chemical shift difference $\Delta = \nu_0^1 - \nu_0^2$.

A key realization is that in the strongly coupled regime ($J_{12} \gg \Delta$), H_{Δ} should be understood as the *driving interaction*, whereas H_J plays the role of the strong *modulating term* that appears in pulse sequences as a matching condition.

Suppose that the SLIC sequence is defined as follows:

$$\mathrm{SLIC}_{\pm}(\tau) = (\pi/2)_{-\mathrm{y}} - [\mathrm{SL}]_{\pm\mathrm{x}}^{\omega_{\mathrm{nut}}=\omega_{\mathrm{J}}}(\tau) - (\pi/2)_{+\mathrm{y}} \tag{6}$$

Where $[\mathrm{SL}]_{\phi}^{\omega_{\mathrm{nut}}=\omega_{\mathrm{J}}}(\tau)$ denotes spin-locking of duration τ, phase ϕ, and nutation frequency ω_J. The final pulse is not necessary at all for the operation of the sequence, but has been inserted to simplify the theory by avoiding working in a tedious tilted frame.

With this considered, we may now evaluate $\tilde{H}_{\Delta}(\tau)$, which is merely the driving interaction (in this case, the chemical shift interaction) expressed in the interaction frame Hamiltonian of the SLIC sequence, containing the synchronised action of the J-coupling Hamiltonian H_J and a matched rf field $\pm\omega_J I_x$. Considering both positive $(+x)$ and negative $(-x)$ senses of continuous-wave irradiation, we calculate:

$$\begin{aligned}\tilde{H}_{\Delta}(\tau) &= R_y(\pi/2)\exp\left[+i(H_J \pm \omega_J I_x)t\right] H_{\Delta} \exp\left[-i(H_J \pm \omega_J I_x)t\right] R_y(-\pi/2) \\ &= \mp\sqrt{2}\pi\Delta \left(I_x^{S_0,T_{\pm}} - I_{\omega_J \tau}^{S_0,T_{\mp}} \right)\end{aligned} \tag{7}$$

Where we have defined the rotation operator as $R_{\phi}(\beta) = \exp\left[-i\frac{\pi}{2} I_{\phi}\right]$. We can now proceed to calculate the effective Hamiltonian $\overline{H}_{\mathrm{SLIC}_{\pm}}^{(1)}$ over a cycle corresponding to a full J-period $T = 1/J$:

$$\begin{aligned}\overline{H}_{\mathrm{SLIC}}^{(1)} &= \frac{1}{T}\int_0^T \tilde{H}_{\Delta}(\tau)\,d\tau,\ T = 1/J \\ &= \mp\sqrt{2}\pi\Delta I_x^{S_0,T_{\pm}}\end{aligned} \tag{8}$$

The most critical point here is that the mechanism of the SLIC sequence can be fully explained by the above effective Hamiltonian, which engineers a transition between the state $|S_0\rangle$ and either of the triplet states $|T_{\pm}\rangle$, depending on the phase of the SLIC pulse. We now proceed to "translating" this picture to other applications of SLIC.

C. Other contexts where SLIC (and cSLIC) are applicable

Rather than derive multiple effective Hamiltonians for the various different situations in which SLIC (and analogous sequences such as NOVEL) are applicable on a case-by-case basis, we find it more convenient to provide a table containing a list of relevant transitions $|r\rangle \leftrightarrow |s\rangle$, rf amplitude matching conditions (or resonances) in terms of the modulation frequency Ω_μ, and driving terms characterized by a Rabi frequency ω_u.

Sequence	Transition	Matched rf amplitude (ω_{rf})	Driving term [Rabi frequency, ω_μ]	Modulating term [Resonant frequency, Ω_μ]
SLIC (AB)	$\|S_0\rangle \leftrightarrow \|T_\pm\rangle$	ω_{nut}^{12}	chemical shift difference [$\sqrt{2}\pi\Delta$]	J-coupling [$2\pi J_{12}$]
SLIC (PHIP)	$\|\alpha^{\text{S}} S_0^{\text{I}}\rangle \leftrightarrow \|\beta^{\text{S}} T_\pm^{\text{I}}\rangle$	ω_{nut}^{S}	differential coupling [$\pi(J_{IS} - J_{I'S})/2$]	homonuclear J-coupling [$2\pi J_{II}$]
SLIC (AA'XX')	$\|T_\pm^A S_0^X\rangle \leftrightarrow \|S_0^A T_0^X\rangle$	ω_{nut}^{A}	out-of-pair coupling difference [$\pi(J_{AX} - J_{AX'})/\sqrt{2}$]	in-pair coupling (difference) [$2\pi(J_{AA} - J_{XX'})$]
SLIC (AA'XX')	$\|T_\pm^A T_0^X\rangle \leftrightarrow \|S_0^A S_0^X\rangle$	ω_{nut}^{A}	out-of-pair coupling difference [$\pi(J_{AX} - J_{AX'})/\sqrt{2}$]	in-pair coupling (sum) [$2\pi(J_{AA} + J_{XX'})$]
NOVEL	$\|\alpha^{\text{e}}\beta^{\text{n}}\rangle \leftrightarrow \|\beta^{\text{e}}\alpha^{\text{n}}\rangle$ or $\|\alpha^{\text{e}}\alpha^{\text{n}}\rangle \leftrightarrow \|\beta^{\text{e}}\beta^{\text{n}}\rangle$	$\omega_{\text{nut}}^{\text{e}}$	elecron-nuclear coupling [$\pi J_{\text{en}}/2$]	nuclear Larmor frequency [ω_0^{n}]

Table SI. A table of various analogues of the SLIC pulse sequence. All sequences are fully characterized by their particular target transitions, the matching condition $\omega_{\text{rf}} = \Omega_\mu$ relating a nutation frequency to the modulation frequency Ω_μ, and driving terms which allow population transfer with a Rabi frequency ω_μ.

D. Generalized response of SLIC to rf amplitude errors

In the most general case, the dependence of the excitation efficiency of SLIC on rf amplitude errors $\varepsilon_{\mathrm{rf}}$ corresponds to the familiar textbook case of a detuned Rabi oscillation:

$$\xi_{\mathrm{SLIC}}(t) = \sin^2(\theta_\mu)\sin^2\left(\frac{1}{2}\omega_\mu \csc(\theta_\mu) t\right) \tag{9}$$

Where the "detuning angle" θ_μ is defined in terms of the Rabi frequency ω_μ and the effective detuning frequency Ω_μ:

$$\theta_\mu = \arctan\left(\frac{\omega_\mu}{\Omega_\mu \varepsilon_{\mathrm{rf}}}\right) \tag{10}$$

For a nominal duration $t = \pi/\omega_\mu$ the efficiency is simply:

$$\xi_{\mathrm{SLIC}}(\pi/\omega_\mu) = \frac{\pi^2}{4}\mathrm{sinc}^2\left(\frac{\pi}{2}\csc(\theta_\mu)\right) \tag{11}$$

Which is a narrowband sinc-squared response, depicted in Figure S1. The extreme sensitivity of the SLIC sequence (and analogues such as NOVEL) to the matching condition presents a major source of inconvenience to the variety of experiments mentioned beforehand.

E. The effective Hamiltonian of the cSLIC sequence in an AB spin system

The effective Hamiltonian of the cSLIC sequence, as shown in the appendix of another reference[5], is given by the following in the limit of a negligibly short compensating pulse ($\alpha \to 1$):

$$\begin{aligned}\overline{H}^{(1)}_{\mathrm{cSLIC}} &= \lim_{\alpha \to 1}\int_0^{1/J} \tilde{H}_\Delta(t)\,\mathrm{d}t \\ &= -\sqrt{2}\pi\Delta\left[\mathrm{sinc}\,(f_+)\, I_x^{S_0,T_+} - \mathrm{sinc}\,(f_-)\, I_x^{S_0,T_-}\right]\end{aligned} \tag{12}$$

where the arguments of the sinc functions are:

$$\begin{aligned} f_+ &= \pi\varepsilon_{\mathrm{rf}} \\ f_- &= \pi\,(2+\varepsilon_{\mathrm{rf}})\end{aligned} \tag{13}$$

These represent a pair of counter-rotating frequency components, centred at the resonances of the two nominal SLIC matching conditions: $\omega_{\mathrm{nut}}^{\mathrm{SLIC}} = +\omega_J$ ($\varepsilon_{\mathrm{rf}} = 0$), and $\omega_{\mathrm{nut}}^{\mathrm{SLIC}} = -\omega_J$ ($\varepsilon_{\mathrm{rf}} = -2$).

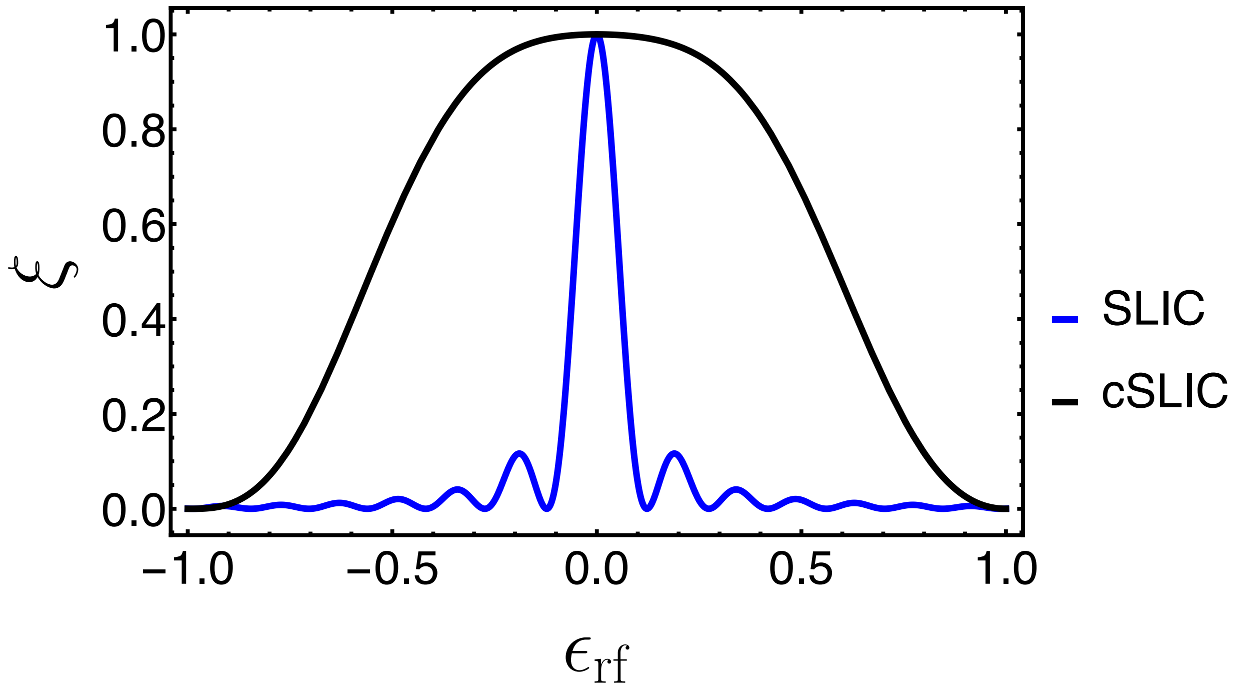

Figure S1. Analytical excitation efficiences ξ of the SLIC and cSLIC sequences as a function of rf amplitude error $\varepsilon_{\mathrm{rf}}$, using equations 11 and 15. The parameters used for SLIC are the Rabi frequency $\omega_\mu = \omega_\Delta/\sqrt{2}$ and $\Omega_\mu = \omega_J$, with $\omega_\Delta = 2\pi \times 10$ and $\omega_J = 2\pi \times 100$.

F. Generalized response of cSLIC as a function of rf amplitude errors

In the most general case, the excitation efficiency of cSLIC is given by the following function:

$$\xi_{\mathrm{cSLIC}}(t) = \frac{f_-^2 - f_+^2}{f_-^2 + f_+^2} \sin^2\left(\frac{1}{2}\omega_\mu \sqrt{\mathrm{sinc}^2(\mathrm{f}_-) + \mathrm{sinc}^2(\mathrm{f}_+)t}\right) \tag{14}$$

When compared to Equation 9, the dependence on the Ω_μ term has vanished. The dependence on $\varepsilon_{\mathrm{rf}}$ is solely due to interference between the two matching conditions f_- and f_+.

For a nominal duration $t = \pi/\omega_\mu$, the excitation efficiency of cSLIC becomes:

$$\xi_{\mathrm{cSLIC}}(t = \pi/\omega_\mu) = \frac{f_-^2 - f_+^2}{f_-^2 + f_+^2} \sin^2\left(\frac{\pi}{2} \sqrt{\mathrm{sinc}^2(\mathrm{f}_-) + \mathrm{sinc}^2(\mathrm{f}_+)}\right) \tag{15}$$

A comparison of the analytical equations describing the rf error dependence of SLIC (equation 11) and cSLIC (equation 15) is shown in Figure S1. The cSLIC sequence (or what would be the analogous "cNOVEL" sequence in the context of DNP) has a broadband response whereas the SLIC sequence has a narrowband response that worsens for larger values of Ω_μ/ω_μ.

II. SUPERCYCLED VARIANTS OF cSLIC

$\mathscr{C}$, the cyclic element building block of the cSLIC pulse sequence used in this paper, can be expressed in terms of the two inversion elements A and B:

$$\begin{aligned} \mathrm{A} &= 180_{x}^{\omega_{\mathrm{nut}}=\omega_J} \\ \mathrm{B} &= 180_{-x}^{\omega_{\mathrm{nut}}=\omega_{\mathrm{strong}}} \end{aligned} \tag{16}$$

The position of the compensating pulse element "BB" is not particularly important; it may be placed at the beginning, middle, or end of the pulse sequence without affecting the performance, and indeed all of the following cyclic elements may be used to compose the cSLIC sequence:

$$\mathscr{C}_1 = \mathrm{AABB} \tag{17}$$

$$\mathscr{C}_2 = \mathrm{ABBA} \tag{18}$$

$$\mathscr{C}_3 = \mathrm{BBAA} \tag{19}$$

The variant of cSLIC used in this paper (ABBA) was chosen purely for aesthetic reasons, and as a tribute to the Swedish pop band that bears the same moniker.

Combining the different permutations C_i can be used to improve the performance of cSLIC as a function of resonance offset, at the expense of increased sensitivity to rf amplitude errors. For example, some "supercycles"[6,7] with better off-resonance performance (denoted S_i) are:

$$\begin{aligned} S_1 &= \mathscr{C}_2 \\ S_2 &= \mathscr{C}_1\mathscr{C}_2 \\ S_3 &= \mathscr{C}_1\mathscr{C}_2\mathscr{C}_3 \end{aligned} \tag{20}$$

The performance of these supercycled variants is shown in Figure S2. The S_3 variant of cSLIC shows more uniform performance, with no generation of negative singlet order, at the expense of a smaller on-resonance $\varepsilon_{\mathrm{rf}}$ bandwidth.

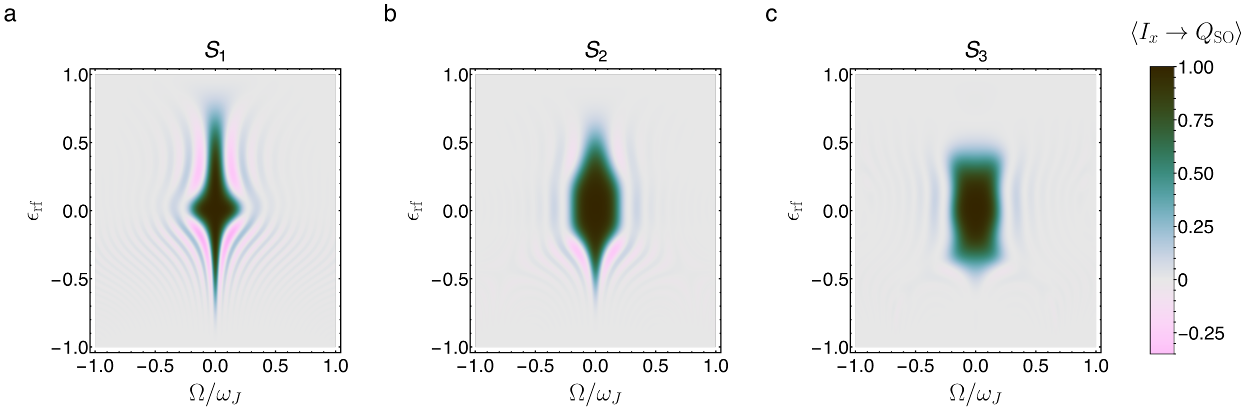

Figure S2. Numerical simulations showing the performance of supercycled variants of cSLIC as a function of rf amplitude errors $\varepsilon_{\mathrm{rf}}$ and relative resonance offset errors Ω/ω_J. Panel (a): the primitive cycle S_1 = ABBA. Panel (b): the 8-step supercycle S_2 = AABBABBA. Panel (c): the 12-step supercycle S_3 = AABBABBABBAA. The simulations are performed for a 2-spin-1/2 system with $\omega_J = 2\pi \times 100$, and $\omega_\Delta = 2\pi \times 3$, assuming $\alpha = 0.99$.

REFERENCES

[1]M. C. Korzeczek, I. Schwartz, and M. B. Plenio, arXiv **2508.07488** (2025), 10.48550/arXiv.2508.07488, arXiv:2508.07488.

[2]N. Wili, A. B. Nielsen, L. A. Völker, L. Schreder, N. C. Nielsen, G. Jeschke, and K. O. Tan, Science Advances **8**, eabq0536 (2022).

[3]A. Wokaun and R. R. Ernst, Journal of Chemical Physics **67**, 1752 (1977).

[4]S. Vega, The Journal of Chemical Physics **68**, 5518 (1978).

[5]U. D. Heramun, M. Sabba, D. Yamano, C. Bengs, B. Legrady, G. Pileio, S. Thompson, and M. H. Levitt, The Journal of Chemical Physics **164**, 064201 (2026).

[6]D. P. Burum, M. Linder, and R. R. Ernst, Journal of Magnetic Resonance (1969) **44**, 173 (1981).

[7]M. H. Levitt, The Journal of Chemical Physics **128**, 052205 (2008).
